# The roles of autonomous motivation and self-compassion on changes in eating disorder pathology across inpatient eating disorder treatment

**DOI:** 10.1007/s40519-026-01890-0

**Published:** 2026-07-23

**Authors:** R. Batchelor, K. Morgan-Lowes, S. J. Egan, J. Howell, C. Thøgersen-Ntoumani

**Affiliations:** 1 The Oxford Institute of Clinical Psychology Training and Research, University of Oxford, Oxford, UK; 2 Discipline of Psychology, School of Population Health, Curtin University, Perth, Australia; 3 enAble Institute, Faculty of Health Sciences, Curtin University, Perth, Australia; 4 Fremantle Mental Health Service, Fremantle, WA Australia; 5 Danish Centre for Motivation and Behaviour Science (DRIVEN), Institut for Idræt Og Biomekanik, Department of Sports Science and Clinical Biomechanics, Syddansk Universitet, Odense, Denmark; 6 School of Sport, Exercise and Rehabilitation Sciences, University of Birmingham, Birmingham, UK

**Keywords:** Eating disorders, Anorexia nervosa, Self-compassion, Autonomous motivation

## Abstract

**Purpose:**

Autonomous motivation and self-compassion may potentially influence outcomes in eating disorder treatment. Self-compassion may reduce criticism and shame, which have been implicated in the maintenance of anorexia nervosa. This study examined whether baseline autonomous motivation and self-compassion, and their interaction, were associated with changes in eating disorder symptoms in women with anorexia nervosa receiving inpatient treatment.

**Methods:**

Participants were 56 women (*M* age = 23.8, range = 16–52 years) diagnosed with anorexia nervosa and admitted to an inpatient eating disorder programme. Eating disorder symptoms (Eating Disorder Examination-Questionnaire; EDE-Q), autonomous motivation (Autonomous and Controlled Motivation for Treatment Questionnaire; ACMTQ), and self-compassion (Self-Compassion Scale-Short Form (SCS-SF) were measured at pre- and post-treatment.

**Results:**

Latent change score models indicated that higher baseline autonomous motivation was associated with greater reductions in eating disorder symptoms across treatment. Baseline self-compassion and the interaction between autonomous motivation and self-compassion were not associated with changes in eating disorder symptoms. Post hoc Monte Carlo simulations indicated that the models were adequately powered to detect effects of the observed magnitude.

**Conclusion:**

The findings are consistent with Self-Determination Theory in eating disorder populations and suggest that higher autonomous motivation is associated with greater reductions in eating disorder symptoms. Future research should examine whether interventions targeting autonomous motivation may be associated with improved treatment outcomes in women with anorexia nervosa.

**Level of evidence:**

Level III: Evidence obtained from well-designed cohort or case–control analytic studies.

## Background

Ambivalence towards change and motivation for treatment is a common challenge in eating disorder treatment [1, 2]. Lower levels of autonomous motivation have been associated with poorer eating disorder treatment outcomes and potentially higher treatment attrition rates [3–5]. A theoretical framework that may help explain motivation towards change in eating disorders is Self-Determination Theory [6]. Although originally developed as a general theory of human motivation, Self-Determination Theory has increasingly been applied to eating disorders. Indeed, autonomous forms of motivation and satisfaction of basic psychological needs have been linked to more adaptive eating regulation, lower eating disorder symptom severity, and improved treatment engagement [7, 8].

Self-Determination Theory proposes that motivation exists along a continuum, ranging from amotivation, characterised by a lack of intention to act, through to controlled motivation, in which behaviour is driven by external pressures such as expectations of others, guilt, or shame [6]. At the other end of the continuum is autonomous motivation, where actions are guided by personal values, commitment, or enjoyment. Within clinical eating disorder populations, higher autonomous motivation for treatment has been associated with better outcomes. For example, higher pre-treatment autonomous motivation has been associated with lower eating disorder symptoms at post-treatment in both inpatient and outpatient samples [9–12]. Further, adults with higher autonomous motivation during inpatient and outpatient treatment demonstrate significantly faster reductions in eating disorder symptoms compared to those with lower autonomous motivation [11, 12].

In contrast, controlled motivation for treatment has not been associated with eating disorder symptoms or treatment response in clinical eating disorder populations receiving inpatient or outpatient treatment [10–12]. This may indicate that externally driven reasons for treatment are less strongly related to symptom improvement, or that the effects of controlled motivation vary according to treatment context and how motivational constructs are measured. Identifying psychological factors associated with symptom change may help inform treatment stratification and the development of interventions that target modifiable mechanisms, such as motivation, to improve treatment engagement and outcomes.

According to Self-Determination Theory, an individual’s motivation type can be influenced by a range of interpersonal and intrapersonal factors [6]. One intrapersonal factor that may be associated with autonomous motivation is self-compassion [13]. Self-compassion is a multifaceted concept in which individuals relate to themselves with kindness, acceptance, and non-judgement during times of difficulty [13]. Lower self-compassion has been associated with higher symptoms of anxiety, depression, disordered eating and clinical eating disorders [14–16].

Within a cognitive-interpersonal maintenance model of anorexia nervosa [17], high levels of self-criticism, shame, and interpersonal avoidance are proposed to maintain eating disorder symptoms and hinder engagement in recovery processes. Consistent with this framework, self-compassion may be conceptualised as a potential protective factor associated with lower self-criticism and shame, and may support more open engagement with treatment and recovery-oriented behaviours [18, 19]. Through its associations with lower self-criticism and shame and a more accepting and supportive stance towards oneself, self-compassion may be associated with greater openness to treatment and engagement with recovery goals that are experienced as personally meaningful rather than driven primarily by external pressure or fear of failure.

Anorexia nervosa is a particularly relevant disorder in which to examine autonomous motivation, as symptoms are often experienced as ego-syntonic and closely intertwined with an individual’s sense of identity and ambivalence towards recovery [20]. Individuals may simultaneously recognise the harmful consequences of the illness while perceiving the eating disorder as serving valued functions, such as providing a sense of control, achievement, or self-worth [21]. This conflict may make autonomous motivation especially important, as sustained engagement in treatment may depend on the extent to which recovery is experienced as personally meaningful and aligned with broader life values [22].

It has been proposed that higher self-compassion may support autonomous motivation by fostering a less self-critical relationship with oneself, which may be associated with greater internalisation of treatment goals and the pursuit of proactive, values-consistent behaviours that enhance well-being [13, 23]. In the context of Self-Determination Theory, self-compassion may be associated with greater engagement in recovery goals when these goals are experienced as personally meaningful and aligned with an individual’s values, rather than driven primarily by external pressure, guilt, or fear of failure. In a university sample, Hope et al. [24] found that self-compassion moderated the association between autonomous motivation and negative affect, where autonomous motivation was related to lower negative affect for students high in self-compassion. As such, the authors theorised that for students who are highly self-compassionate, pursuing autonomously motivated goals may be particularly beneficial in promoting well-being.

While a meta-analysis by Turk and Waller [15] showed lower self-compassion to be associated with greater eating disorder symptom severity across clinical and non-clinical populations, only one study to date has examined the relationship between autonomous motivation and self-compassion in individuals diagnosed with eating disorders. Carter and Kelly [11] reported that participants receiving inpatient or outpatient eating disorder treatment who endorsed higher self-compassion at admission also reported higher autonomous motivation for entering treatment.

It remains unclear how autonomous motivation and self-compassion are associated with eating disorder symptoms across treatment in clinical eating disorder populations, and whether either variable moderates changes in eating disorder symptoms across treatment. Further investigation of these relationships may enhance understanding of individual differences in treatment response and inform the development of interventions aimed at improving treatment outcomes via a focus on motivation. Notably, individuals with anorexia nervosa restricting subtype have been shown to report lower baseline autonomous motivation compared with those with binge–purge presentations or bulimia nervosa [11], highlighting anorexia nervosa as a particularly relevant diagnostic group in which to examine autonomous motivation.

The aim of this study was to examine associations between baseline autonomous motivation and self-compassion and changes in eating disorder symptoms for women diagnosed with anorexia nervosa across inpatient eating disorder treatment. It was hypothesised that higher baseline self-compassion and autonomous motivation would be associated with greater reductions in eating disorder symptoms from pre- to post-treatment, controlling for baseline eating disorder symptoms, body mass index (BMI), age, and treatment duration.

A second aim was to examine whether there was an interaction between baseline self-compassion and autonomous motivation in predicting change in eating disorder symptoms across treatment. Given the limited empirical evidence examining the joint influence of these variables in eating disorder populations, this analysis was considered exploratory. It was hypothesised that the interaction between self-compassion and autonomous motivation would predict changes in eating disorder symptoms, such that self-compassion would be more strongly associated with symptom reductions at higher levels of autonomous motivation.

## Method

### Participants

**Fig. 1:**
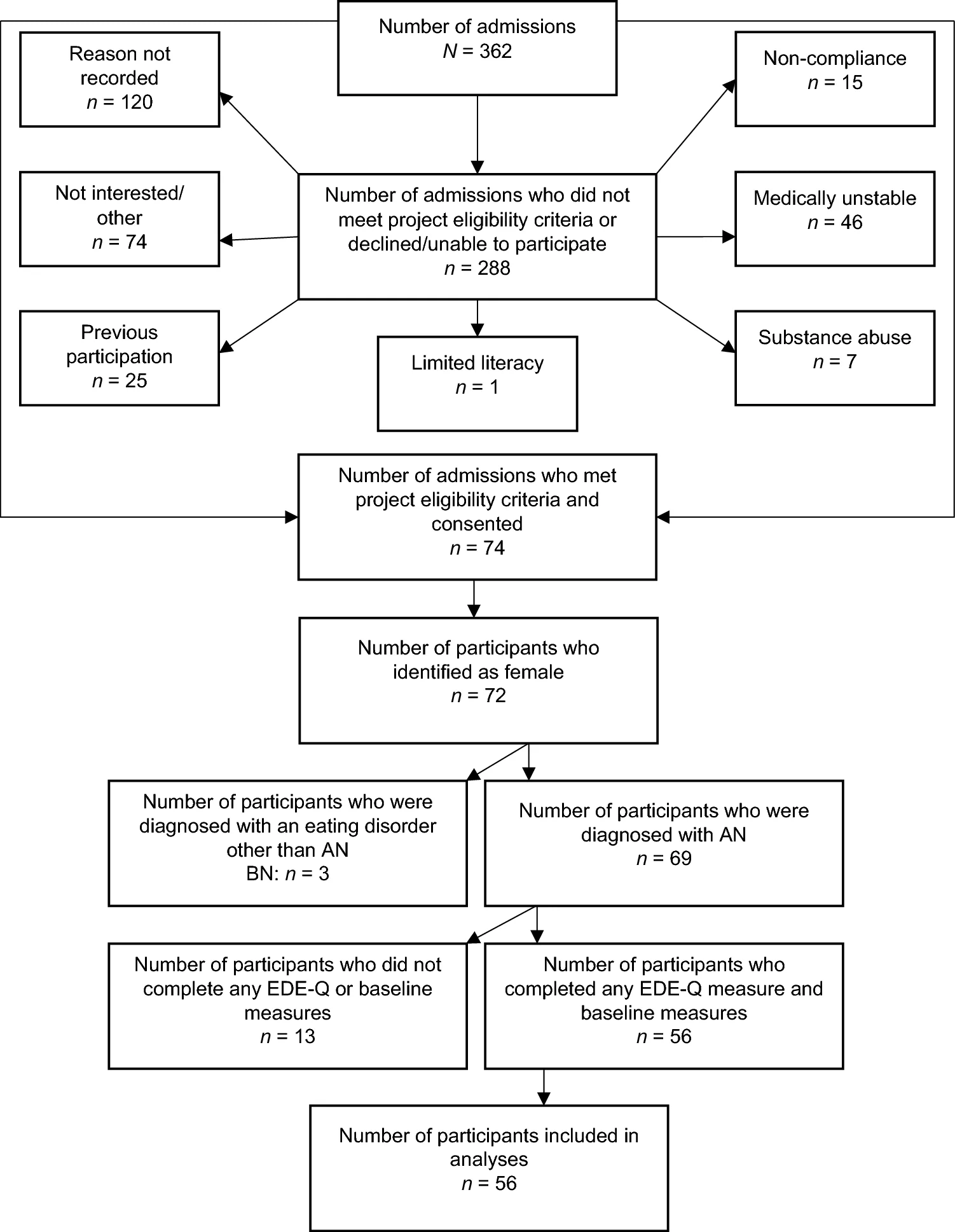
Project recruitment and study engagement. This diagram depicts the recruitment of potential participants who were admitted to the hospital inpatient eating disorder programme between June 2016 and November 2019. It further shows the flow of participants eligible for analyses. AN: anorexia nervosa; BN: bulimia nervosa; EDE-Q: Eating Disorder Examination-Questionnaire

Participants were recruited from patients admitted to an inpatient eating disorder programme, Hollywood Hospital, based in Western Australia, between June 2016 and November 2019. Inclusion criteria were: (a) provision of informed consent (and caregiver consent for participants under 18 years); (b) a primary diagnosis of anorexia nervosa diagnosed by a psychiatrist through a clinical interview using the Diagnostic and Statistical Manual of Mental Disorders-5 criteria (DSM-5) [25] and (c) identify as female. Exclusion criteria were: (a) another clinical eating disorder diagnosis (i.e. bulimia nervosa); (b) active substance abuse; (c) inability/unwillingness to use an iPad for data collection; and (d) previous participation in the study, given the possibility of multiple inpatient admissions. Recruitment flow and study engagement are presented in Fig. 1.

Participants were 56 females aged between 16 and 52 years (*M* = 23.8, *SD* = 7.3). The mean duration of inpatient treatment at the clinic was 27 days (*SD* = 12.8, range = 6–56 days). The diagnoses in the sample were 82% anorexia nervosa restricting subtype (*n* = 46) and 18% anorexia nervosa binge/purge subtype (*n* = 10). Given the small number of participants in the binge/purge subgroup, analyses were conducted on the combined sample and subtype-specific comparisons were not performed. The mean self-reported duration of an individual’s eating disorder was 6.9 years (*SD* = 6.8, range = 1–37 years). Mean BMI at admission was 17.9 (*SD* = 2.2), and ranged from 13.3 to 23.5.

### Measures

The following measures were administered pre- and post-treatment during the pre-discharge appointment (at point of discharge).

#### Autonomous motivation for treatment

Autonomous motivation for treatment was assessed using the Autonomous and Controlled Motivation for Treatment Questionnaire (ACMTQ), a 12-item measure comprising two subscales assessing autonomous and controlled motivation [26]. The ACMTQ has been adapted from the original version by changing language to be suitable for use in eating disorder populations [9, 11], and has shown acceptable psychometric properties, including reliability and construct validity, in prior eating disorder samples [9, 11]. Items are rated on a 7-point Likert scale ranging from 1 (strongly disagree) to 7 (strongly agree), with higher scores indicating greater endorsement of the motivation type. Subscale scores were calculated by summing relevant items. Only the autonomous motivation subscale was analysed in the present study. Internal consistency at baseline was good (*α* = 0.89).

#### Self-compassion

Self-compassion was measured using the Self-Compassion Scale-Short Form [27], a 12-item version of the original Self-Compassion Scale [28], which has been supported as valid and reliable among people with eating disorders [29]. The measure includes six positively worded and six negatively worded items rated on a 5-point Likert scale from 1 (almost never) to 5 (almost always). Negative items were reverse scored, and overall mean scores were calculated, in line with the measure’s scoring instructions and clinical eating disorder samples in previous research [16, 27], with higher scores indicating greater self-compassion. Internal consistency at baseline was good (*α* = 0.86).

#### Eating disorder symptoms

The Eating Disorder Examination-Questionnaire (EDE-Q 6.0), a 28-item self-report measure evaluating behavioural and psychological features of eating disorders over the past 28 days [30] was used. The EDE-Q has been supported as valid and reliable among clinical eating disorder samples including anorexia nervosa [31]. Items assessing psychological features are rated on a 7-point Likert scale ranging from 0 (no days/not at all) to 6 (every day/markedly). The EDE-Q yields four subscales—weight concern, shape concern, eating concern, and dietary restraint—the mean of which provides a overall (global) score of eating disorder severity. The EDE-Q overall (global) score was used in its original form as a continuous measure of eating disorder symptom severity, consistent with common practice in eating disorder research [31]. Clinical interpretation based on cut-off scores was not undertaken because the study focused on associations between study variables and changes in symptom severity over time within a clinically diagnosed inpatient sample, rather than on categorising participants according to symptom severity thresholds. Higher scores indicate greater symptom severity. Internal consistency for the overall (global) score at baseline was acceptable [21] (*α* = 0.77).

### Procedure

This study was part of a larger unpublished project that used an ecological momentary assessment framework [32] where participants were assessed across inpatient eating disorder treatment; however, only pre- and post-treatment data were included in the present study. Participants were recruited by clinical staff on admission to the inpatient eating disorder programme at Hollywood Hospital. The programme is delivered by a multidisciplinary team and provides specialised eating disorder treatment including medical stabilisation, individual therapy, group cognitive behavioural therapy, individual dietetic support, and meal support. Treatment duration varied according to individual clinical needs.

Patients entering inpatient treatment for an eating disorder were invited to participate on admission by clinical staff and provided with an information sheet outlining the study upon initial presentation to the unit. Recruitment followed a consecutive admissions approach, whereby all newly admitted eligible patients during the recruitment period were considered for participation; however, individuals who had previously been approached during an earlier admission were not re-approached on subsequent admissions. No additional clinical screening or selection criteria were applied by staff beyond eligibility for inpatient admission and capacity to provide informed consent.

To minimise potential power dynamics associated with recruitment by clinical staff within an inpatient setting, patients were explicitly informed that the study was being conducted by external university researchers and not by their treating clinical team, and that participation was entirely voluntary. It was clearly emphasised that declining participation or withdrawing at any point would not affect their treatment, care, or relationship with staff in any way. Overall participation rates were relatively low in relation to the total inpatient population, further suggesting that recruitment did not exert undue coercive pressure.

Recruitment typically occurred on the first day or after a few days if the individual felt overwhelmed (*M* = 0.40 days after admission, *SD* = 0.80, range = 0–3). A sensitivity analysis of the inclusion of this time-lag showed no impact on the current findings and is available on the Open Science Framework: https://osf.io/qftb5/overview. As compensation for their time, coffee vouchers for a local café were given to participants. Following consent, participants were provided with an iPad containing questionnaires for completion. BMI and EDE-Q data were collected as part of routine clinical practice at admission and at point of discharge (during the pre-discharge appointment), with BMI additionally assessed weekly by clinical staff (weekly assessments were not considered in the analyses of this study). The ACMTQ and SCS-SF were administered at pre- and post-treatment (at point of discharge). Automated prompts indicated when questionnaires were available for completion.

### Data analysis

The hypotheses were tested using latent change score (LCS) models [33, 34] in Mplus Version 8.5 [35]. LCS models construct the amount of change over time as a latent variable, which can then be treated as an outcome to be predicted. LCS models with two timepoints (and no covariates) are statistically identical to a paired samples *t*-test, and evaluating relationships with the amount of change over time only requires estimating a single parameter per covariate [36]. This simplicity makes the LCS model a practical choice for analyses of change in small samples. Missing data were handled using full-information maximum-likelihood (FIML), which has been shown to be preferable in longitudinal analyses with small samples [37].

**Fig. 2:**
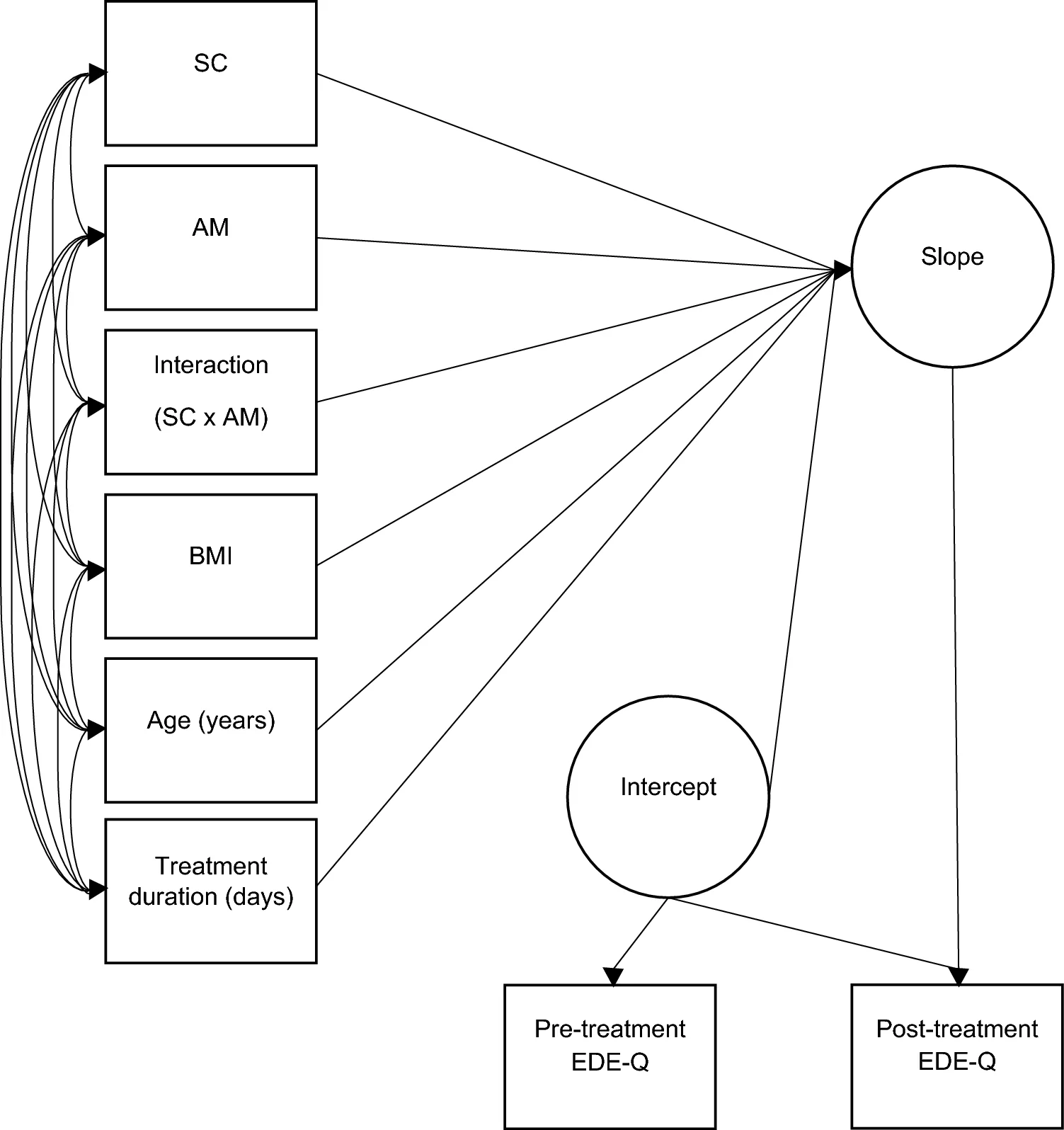
Latent change score model predicting changes in eating disorder symptoms. This path analysis model shows the baseline variables predicting changes in eating disorder symptoms scores from pre- to post-treatment. Baseline predictors of interest were self-compassion, autonomous motivation, and the interaction between the two. BMI, age, and treatment duration were entered as control variables. AM: autonomous motivation; BMI: body mass index; EDE-Q: Eating Disorder Examination-Questionnaire; SC: self-compassion; interaction: self-compassion x autonomous motivation

A separate LCS model for each outcome was estimated, with self-compassion, autonomous motivation, and the (observed) interaction between them entered as predictors (i.e. autonomous motivation x self-compassion; variables were mean-centred), controlling for baseline eating disorder symptoms, BMI, age, and treatment duration (Fig. 2).

Bias-corrected bootstrapped confidence intervals were calculated using 10,000 bootstrap samples. Post hoc power analyses using Monte Carlo simulation were conducted to assess the sensitivity of the estimates to the small sample size, evaluating how consistently they could be recovered across repeated simulation. All analysis inputs and outputs are available on the Open Science Framework: https://osf.io/qftb5/overview.

To examine moderation effects, standardised loop plots were generated in Mplus. These plots depict how the association between predictors and outcome changes across values of the moderator, ranging from −3 to +3 standard deviations from the mean, while controlling for baseline eating disorder symptoms, BMI, age, and treatment duration. Loop plots are a continuous extension of the ‘simple slopes’ visualisation. Where ‘simple slopes’ plots the predictor on the X-axis, the outcome on the Y-axis, and the trend line at three values of the moderator (typically mean and ± 1 SD), the loop plot shows the moderator on the X-axis and the predictor–outcome relationship (i.e. trend line) on the Y-axis. Loop plots allow for interpreting the estimated moderating effect at the full range of values of the moderator, as well as showing the confidence intervals for that effect.

## Results

Table 1 presents the means, standard deviations, and zero-order Pearson correlations between self-compassion, autonomous motivation, eating disorder symptoms, BMI, age, and treatment duration. At baseline, self-compassion was positively correlated with autonomous motivation and negatively correlated with eating disorder symptoms, with both associations of a comparable moderate magnitude. Baseline autonomous motivation was positively correlated with age, while BMI was negatively correlated with treatment duration, indicating that lower BMI was associated with longer inpatient treatment duration.

**Table 1:** Means, standard deviations (SD), and zero-order Pearson correlations between baseline study variables

	Variable	Mean	*SD*	1	2	3	4	5	6
1	Self-compassion	2.0	0.6	–					
2	Autonomous motivation	5.3	1.4	.34*	–				
3	Eating disorder symptoms	4.5	1.2	− .34*	− .14	–			
4	Body mass index	17.9	2.2	− .03	.11	.27	–		
5	Age (years)	23.8	7.3	.04	.32*	− .02	− .09	–	
6	Treatment duration (days)	26.7	12.8	.00	− .10	.15	− .42**	− .06	–

*p <.05. **p < .01. ***p < .001

The post hoc power analysis for both the BMI and EDE-Q LCS models identified that the models were sufficiently powered with the current sample size. Table 2 presents the unstandardised beta coefficients, *p* values, and bootstrapped 95% confidence intervals for the LCS models. There were significant negative associations between baseline eating disorder symptoms and the amount of change in eating disorder symptoms across treatment. Higher baseline eating disorder symptoms were associated with greater reductions in symptoms over time, such that each 1-point increase at baseline was associated with approximately a 0.4-point greater decrease across treatment.

**Table 2:** Unstandardised estimates for latent change score models exploring eating disorder symptoms outcomes

Baseline predictors of symptom change	Predicting EDE-Q score			Predicting BMI		
	*B*	*p*	95% CI	*B*	*p*	95% CI
Baseline variable	− 0.42*	.003	[− 0.68, − 0.14]	− 0.24***	< .001	[− 0.34, − 0.15]
SC	0.16	.593	[− 0.47, 0.70]	0.17	.301	[− 0.10, 0.55]
AM	− 0.27*	.007	[− 0.49, − 0.08]	0.03	.560	[− 0.07, 0.16]
SC x AM interaction	0.06	.777	[− 0.39, 0.49]	0.08	.509	[− 0.18, 0.30]
Age	− 0.04	.102	[− 0.08, 0.03]	0.02	.161	[− 0.01, 0.04]
BMI	0.12	.212	[− 0.03, 0.35]			
Treatment duration	0.01	.320	[− 0.01, 0.04]	0.07***	< .001	[0.05, 0.08]
R^2^ estimates:						
Symptom change	0.49	< .001		0.77	< .001	

AM: baseline autonomous motivation; BMI: body mass index; EDE-Q: Eating Disorder Examination-Questionnaire; SC: baseline self-compassion; interaction: baseline self-compassion x baseline autonomous motivation; 95% CI: bootstrapped 95% confidence interval

^*^p < .05. ***p < .001

As shown in Table 2, the amount of change in BMI was not significantly associated with baseline self-compassion or autonomous motivation. Baseline autonomous motivation was the only significant direct predictor of the amount of change across inpatient treatment in eating disorder symptoms. Estimates indicated that higher baseline autonomous motivation was associated with greater improvement in eating disorder symptoms from pre- to post-treatment, such that each 1-point increase corresponded to approximately a 0.3-point greater reduction across treatment.

**Fig. 3:**
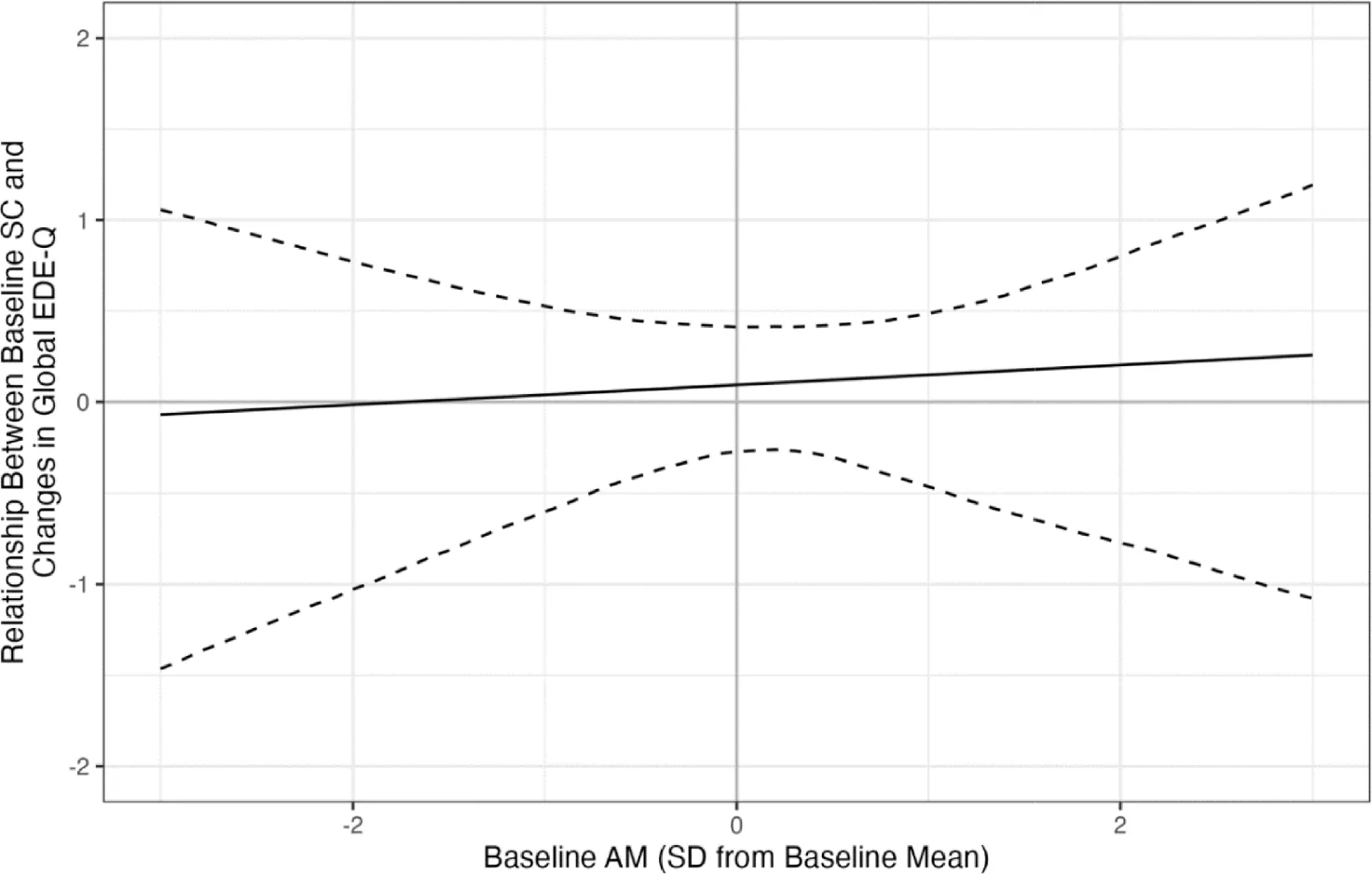
The relationship between baseline self-compassion and changes in eating disorder symptoms moderated by baseline autonomous motivation. The graph depicts the relationship between baseline self-compassion and changes in eating disorder symptoms moderated by baseline autonomous motivation. AM: autonomous motivation; EDE-Q: Eating Disorder Examination-Questionnaire; SC: self-compassion; SD: standard deviation

To examine whether baseline autonomous motivation moderated the association between baseline self-compassion and change in eating disorder symptoms over time, the relationship between baseline self-compassion and symptom change was plotted at varying levels of autonomous motivation (Fig. 3). Confidence intervals for this association crossed zero across all levels of the moderator, indicating no evidence of a moderating effect. R^2^ estimates indicated that approximately 49% of the variance in the change in eating disorder symptoms across time (the LCS change component) were explained by the models.

## Discussion

This study examined associations between self-compassion, autonomous motivation, and their interaction in relation to changes in eating disorder symptoms across inpatient treatment in women with anorexia nervosa. In addition, the potential moderating effects of these predictors on symptom change across treatment were examined.

Higher baseline autonomous motivation was associated with greater reductions in eating disorder symptoms from pre- to post-treatment, partially supporting the first hypothesis and consistent with previous research [9–12]. This finding indicates that individuals who enter inpatient eating disorder treatment with stronger autonomous motivation may experience greater short-term improvements in eating disorder symptoms than those with lower autonomous motivation. Notably, individuals with anorexia nervosa restricting subtype have been shown to report lower baseline autonomous motivation compared with those with binge–purge presentations or bulimia nervosa [11]. In line with this finding, participants in the current study had lower average levels of baseline autonomous motivation when compared to previous research [9–11]. That said, even small increases in autonomous motivation among individuals with the restricting subtype of anorexia nervosa may be associated with clinically meaningful improvements, highlighting the potential value of targeting early motivational enhancement in this group. This interpretation is also consistent with qualitative studies of anorexia nervosa recovery, in which individuals frequently describe the importance of developing a sense of personal agency, autonomy, and ownership over recovery, as well as experiencing treatment relationships as collaborative and autonomy-supportive [22, 38]. Together, these findings suggest that motivation may be particularly relevant when recovery is experienced as personally meaningful rather than externally imposed.

In contrast, baseline self-compassion was not associated with changes in eating disorder symptoms over time. Prior research has suggested that individuals receiving inpatient or outpatient eating disorder treatment who report higher self-compassion at admission also endorse greater autonomous motivation for entering treatment [11]. Accordingly, self-compassion has been theorised to support the development of autonomous motivation [6, 13], which in turn may be associated with eating disorder symptom outcomes; however, the present study did not test mediation or temporal ordering, and therefore these pathways remain speculative. Consistent with this, baseline self-compassion was negatively correlated with eating disorder symptoms at admission, but did not predict change in eating disorder symptoms across treatment.

Inconsistent with the second hypothesis, changes in eating disorder symptoms across treatment were not moderated by baseline self-compassion or autonomous motivation. As such, this may indicate that once autonomous motivation has developed, self-compassion holds less additional utility in explaining reduction in eating disorder symptoms. Alternatively, self-compassion may be associated with the development of autonomous motivation rather than exerting a direct or interactive influence on symptom change, potentially reflecting a broader underlying process not examined in the present study. However, it should also be noted that interaction effects are often more difficult to detect in clinical samples, particularly when sample sizes are modest [39]. Additionally, these interpretations are tentative, as the present study was not designed to examine the temporal ordering or mechanistic relationships among these variables.

These findings have theoretical implications, providing support for the relevance of future research examining the applicability of Self-Determination Theory [6] within eating disorder populations. Specifically, autonomous motivation appears to be an important construct associated with reductions in eating disorder symptoms across inpatient eating disorder treatment for women diagnosed with anorexia nervosa. Clinically, the present findings suggest that higher autonomous motivation at admission is associated with greater reductions in eating disorder symptoms during inpatient treatment, highlighting its potential utility as an indicator of likely treatment response and support needs. These findings do not demonstrate that increasing autonomous motivation will directly improve outcomes; rather, they indicate that autonomous motivation may be a useful marker of treatment response.

Strategies designed to enhance autonomous motivation, such as motivational interviewing or autonomy-supportive communication approaches, may therefore warrant further investigation as potential adjuncts to existing evidence-based interventions. However, recent meta-analytic findings indicate minimal effects of motivational interviewing and motivational enhancement therapy on motivation to change behaviour and eating disorder outcomes, including among individuals with anorexia nervosa in inpatient settings [40]. Accordingly, the current results should be interpreted as providing a rationale for further investigation rather than direct evidence for the effectiveness of these interventions. That said, these findings are constrained by methodological limitations, highlighting the need for larger, well-powered studies with standardised outcome measures and longer-term follow-up. It also highlights the need to examine autonomy-supportive interventions based on Self-Determination Theory, rather than standard motivational interviewing, to determine whether this approach, which has been supported in health psychology and behavioural change interventions [15], is associated with greater self-determined motivation in individuals with anorexia nervosa.

Additionally, the findings provide further insight into the psychological characteristics of individuals with anorexia nervosa who may have greater reductions in eating disorder symptoms over inpatient treatment (i.e. higher autonomous motivation). These results also underscore the potential utility of personalised or adaptive treatment approaches that assess motivation at admission and tailor intervention strategies to individual needs, which may improve treatment response and reduce attrition. Consequently, future research should examine whether providing individuals with anorexia nervosa who report lower autonomous motivation with specific treatment modules to enhance self-determined motivation based on Self-Determination Theory approaches [15] could complement existing evidence-based approaches (e.g., cognitive behaviour therapy-enhanced) [41] to improve treatment outcomes.

### Strengths and limitations

This study has several strengths, including the use of latent change score modelling to robustly examine symptom change across inpatient treatment. The inclusion of a clinically diagnosed inpatient sample enhances clinical relevance, while post hoc Monte Carlo power analyses increase confidence in the interpretation of findings.

However, several limitations warrant consideration. The relatively small sample size may have reduced our ability to detect smaller effects, particularly the hypothesised interaction (i.e. moderation effect) between autonomous motivation and self-compassion. Although participants were recruited over a three-and-a-half-year period, only approximately 19% of admitted patients consented to participate, and not all participants completed post-treatment assessments. This raises the possibility of selection bias, particularly given the study’s focus on motivation, as individuals who consented to participate and completed the study may have been more motivated to engage in treatment and research than those who declined or withdrew. These factors may limit the generalisability of the findings. The inclusion of only female participants further constrains generalisability to other genders. The imbalance in anorexia nervosa subtypes, with a small binge/purge subgroup, also limited the feasibility of subtype-specific analyses and may further constrain the generalisability of findings across diagnostic presentations.

The structured nature of inpatient treatment may also have reduced variability in motivation and symptom change, potentially attenuating observed associations and limiting the extent to which these findings generalise to outpatient or less intensive treatment settings, where greater variability in motivation, treatment engagement, and symptom trajectories may be present. In addition, motivation may be influenced by unmeasured factors consistent with Self-Determination Theory, such as perceived autonomy support and interpersonal relationships within treatment, which were not assessed.

Variability in treatment duration and the absence of post-discharge follow-up data further limit conclusions regarding the durability of observed symptom reductions, restricting interpretation to short-term changes during inpatient treatment and precluding conclusions about whether these associations are maintained following discharge or generalise to longer-term recovery outcomes. Finally, the study design may not have captured important within-person or day-to-day associations; future research using intensive longitudinal methods may help clarify the dynamic relationships between self-compassion, autonomous motivation, and eating disorder outcomes.

## Conclusion

In summary, higher baseline autonomous motivation, but not self-compassion, was associated with greater reductions in eating disorder symptoms across inpatient treatment for women with anorexia nervosa. Given the limitations of the current study, including the small and limited sample, continued investigation of the dynamic interplay between autonomous motivation and self-compassion in eating disorder populations is warranted. Future research should examine whether interventions designed to enhance autonomous motivation and self-compassion are associated with improved treatment outcomes in individuals with anorexia nervosa in an inpatient setting.

### What is already known about this subject?

Previous research has shown that higher autonomous motivation for treatment is associated with better eating disorder treatment outcomes in both inpatient and outpatient settings. Lower self-compassion has also been consistently associated with greater eating disorder symptom severity in both clinical and non-clinical populations. However, it remains unclear how baseline autonomous motivation and self-compassion are associated with changes in eating disorder symptoms across inpatient treatment, and whether they interact in predicting symptom change in clinical inpatient samples.

### What this study adds?

This study shows that higher baseline autonomous motivation, but not self-compassion, is associated with greater reductions in eating disorder symptoms across inpatient treatment for women with anorexia nervosa. In addition, baseline autonomous motivation and self-compassion did not moderate changes in eating disorder symptoms over time. These findings are consistent with the relevance of Self-Determination Theory-related constructs in anorexia nervosa and suggest that future research should examine whether interventions targeting autonomous motivation at inpatient treatment entry are associated with improved treatment outcomes.

## Data Availability

The data are available upon reasonable request and subject to institutional approvals.
